# Management of Giant Retinal Tear with microincision vitrectomy and metallic retinal tacks fixation-a case report

**DOI:** 10.1186/s12886-018-0938-4

**Published:** 2018-10-22

**Authors:** Yo-Chen Chang, Li-Yi Chiu, Tzu-En Kao, Wen-Hsin Cheng, Ting-An Chen, Wen-Chuan Wu

**Affiliations:** 1 Department of Ophthalmology, Kaohsiung Medical University Hospital, Kaohsiung, Taiwan; 2 Department of Ophthalmology, School of Medicine, Kaohsiung Medical University, Kaohsiung, Taiwan

**Keywords:** Giant retinal tear, Microincision vitrectomy surgery, Retinal tack, Wide-angle viewing systems

## Abstract

**Background:**

Giant retinal tear is usually challenging among retinal detachment with recurrent rate up to 45%. Here we presented a case of giant retinal tear being treated by microincision vitrectomy and retinal tacks fixation.

**Case presentation:**

A 53-year-old male presented to our hospital with blurred vision of his right eye for one week with floaters and obscured sensation over nasal visual field. Ocular examination showed a 120 degree giant tear with large inverted flap and retinal detachment of his right eye. The BCVA was only naming digit. Under the impression of giant retinal tear with retinal detachment, 23-gauge pars plana vitrectomy were performed using Constellation high speed vitrectomy system and Topcon non-contact wide angle viewing system. During surgery, the vitreous was removed and perfluorocarbon liquids (PFCL) was injected to help unfolding the large inverted retinal flap. Three retinal tacks were applied to help fixating the large inverted retinal flap. Then, fluid-gas exchange, endolaser photocoagulation and intraocular silicone oil tamponade were performed as well. Initial reattachment of his right retina was achieved and his best corrected visual acuity improved to 0.3 of his right eye postoperatively. There was no recurrent retinal detachment during follow up period of 19 months.

**Conclusions:**

Primary microincision vitrectomy using wide-angle viewing system with intraoperative perfluorocarbon liquids (PFCL) assistant, retinal tacks fixation and intraocular silicone oil tamponade appears to be safe and feasible for managing giant retinal tear with retinal detachment.

## Background

Giant retinal tear (GRT) is usually challenging among retinal detachment with recurrent rate up to 45% [1]. In order to manage such cases, the use of retinal tacks for the repair of complex retinal detachment such as GRT was introduced by Dr. Ando and Dr. de Juan in the 1980s [2, 3]. Microincisional vitrectomy surgery (MIVS) including 23-and 25-gauge was first reported in 2002 and 2005, which had significant reduction in conjunctival injection and postoperative pain and discomfort [4]. Although MIVS was widely used in managing retinal disease, there are no publications on GRT treated by MIVS and retinal tacks in Taiwan. Besides, Wide-angle viewing systems (WAVs) offers a panoramic view in vitreoretinal surgery and it is extremely useful especially for giant retinal tear because the edges of retinal tear can be well seen in one view [5]. Therefore, the purpose of this case report was to present a case of GRT with RD treated by primary MIVS and retinal tacks with WAVs.

## Case presentation

**Fig. 1:**
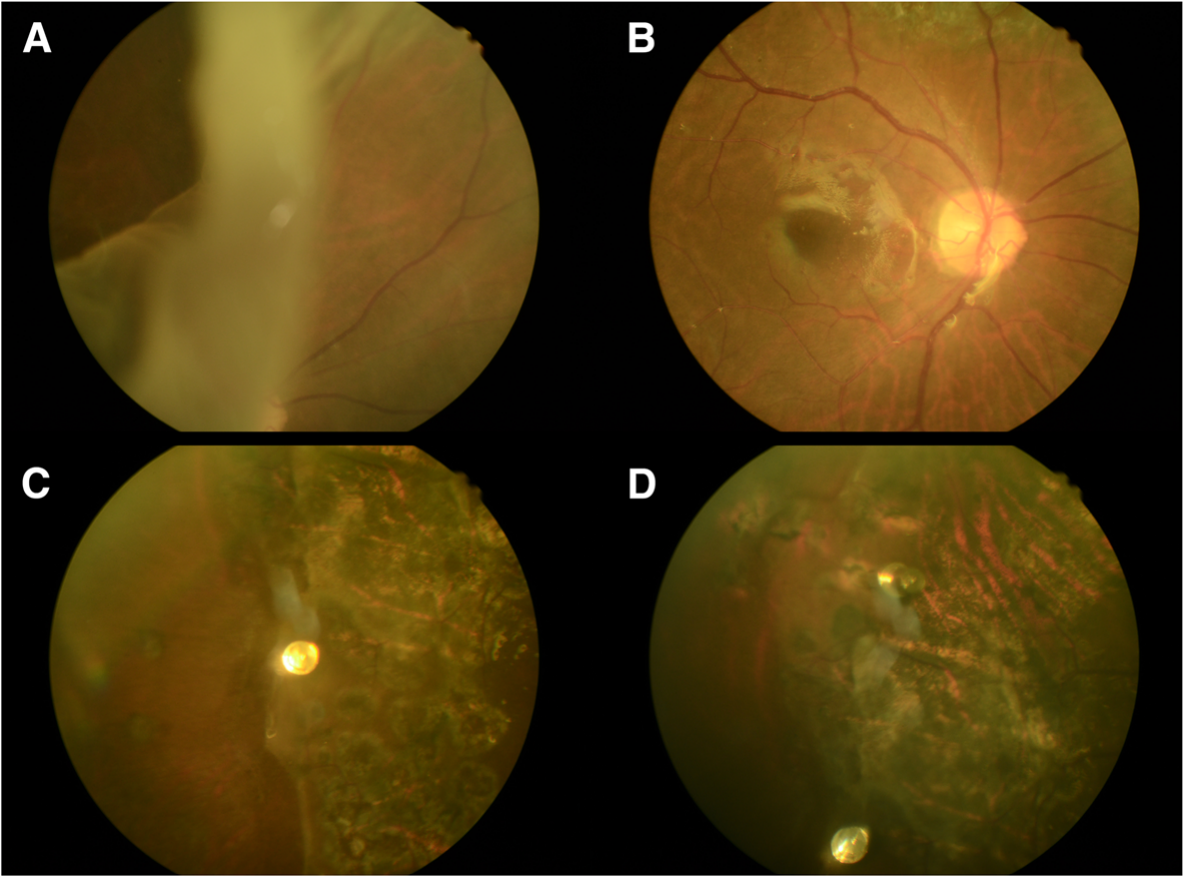
**a.** Fundus examination revealed a 120 degree giant tear with large inverted flap of the 53-year-old man’s right eye. **b, c** and **d.** No recurrent retinal detachment was noted during follow up period and the retina was well fixated by retinal tacks and laser scar

**Fig. 2:**
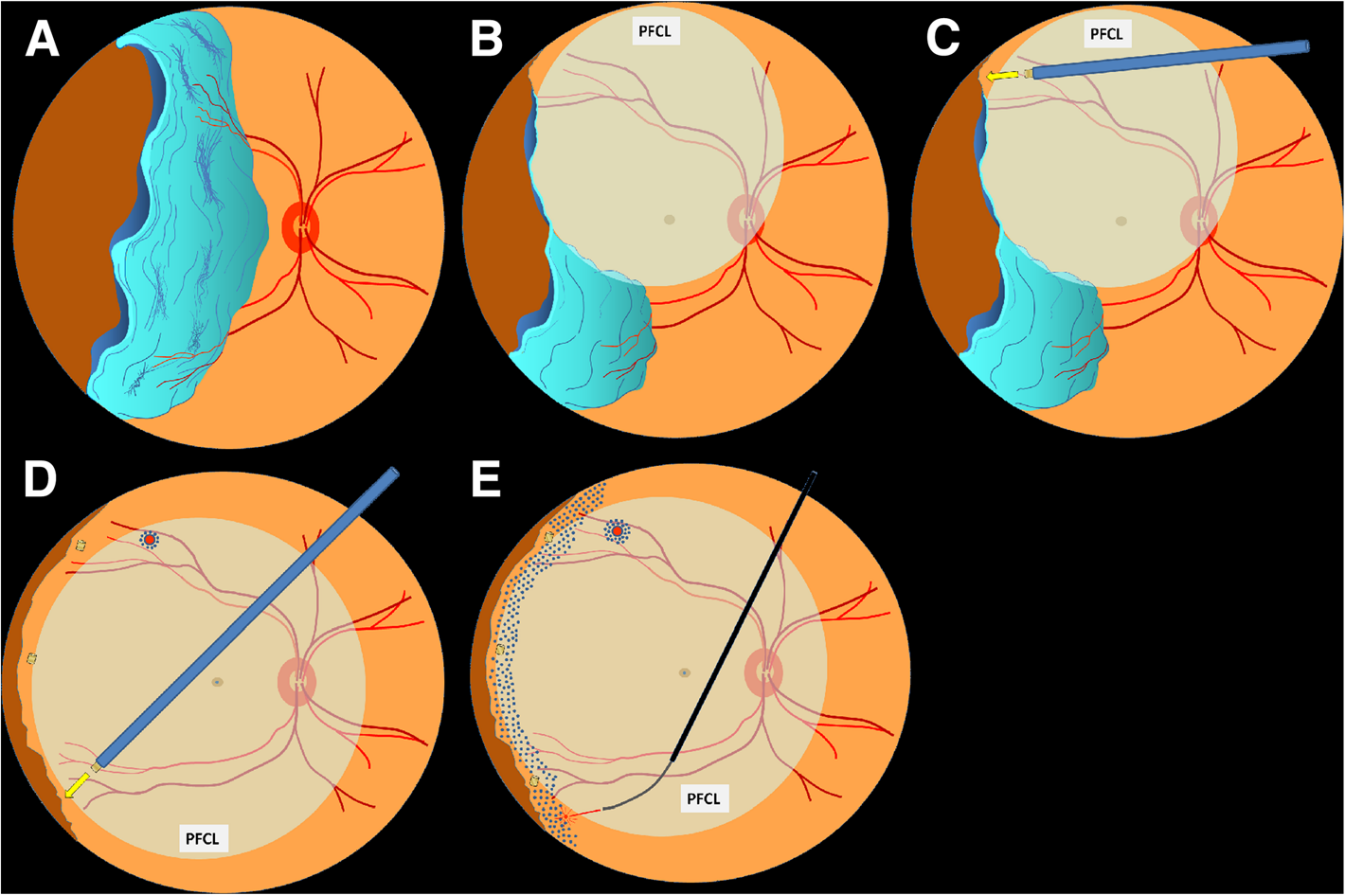
**a.** The temporal side of retina was noted to have a rolled and stiffened edge at the posterior aspect of the giant retinal tear. **b.** Under 23-gauge pars plana vitrectomy, the vitreous was removed and tractions were relieved as much as possible, then perfluorocarbon liquids (PFCL) was injected to help unfolding the large inverted retinal flap. **c** and **d.** Three stainless steel retinal tacks were inserted along the posterior edge of the giant retinal tear using a modified Southerland intraocular forceps to help fixating the large inverted retinal flap. **e.** Fluid-gas exchange, endolaser photocoagulation and intraocular silicone oil tamponade were performed

A 53-year-old male denied past medical and trauma history presented to our hospital with blurred vision of his right eye for one week with floaters and obscured sensation over nasal visual field. Ocular examination showed a 120 degree retinal tear (from 7 to 11 o’clock) with large inverted rigid flap and retinal detachment from 6 to 12 o’clock with macula-off in his right eye (Fig. 1a). The grading of proliferative vitreoretinopathy (PVR) was “Grade C”. The BCVA of his right eye was only naming digit at his first presentation. Under the impression of GRT with retinal detachment, 23-gauge pars plana vitrectomy were performed using Constellation high speed vitrectomy system (Alcon Surgical, Fort Worth, TX, USA) and Topcon non-contact wide angle viewing system (Topcon Medical Inc. Livermore, CA, USA). During surgery, the temporal retina was noted to have a rolled and stiffened edge at the posterior aspect of the giant retinal tear (Fig. 2a). The vitreous was removed and tractions were relieved as much as possible, then perfluorocarbon liquids (PFCL) was injected to help unfolding the large inverted retinal flap (Fig. 2b). Three stainless steel retinal tacks were inserted along the posterior edge of the giant retinal tear using a modified Southerland intraocular forceps [6] to help fixating the large inverted retinal flap (Fig. 2c and d). Then, fluid-gas exchange, endolaser photocoagulation and intraocular silicone oil tamponade were performed as well (Fig. 2e). Initial reattachment of his right retina was achieved and his best corrected visual acuity improved to 0.3 of his right eye postoperatively. There was no recurrent retinal detachment during follow up period of 19 months (Figs. 1b and c).

## Discussion

Previous studies had reported that primary success rate for GRT treated by 20-gauge PPV using PFCL assistant without scleral buckling can achieve over 90% [7]. Another case series reported by Kunikata also suggested similar results for the primary success rate of managing GRT via MIVS [4]. However, treating patients with a rigid inverted retinal flap is relative difficult and retinal tack might make the surgical procedure easier. To date, there’s no similar reports published in Taiwan currently. We performed MIVS for a case of GRT with retinal detachment. To remove the peripheral vitreous completely and release all possible vitreous traction without causing more damage to the retina, we use WAVs and high cutting rate vitrectomy system. Besides, for GRT with large inverted and rigid flap, we use retinal tacks to stabilize the flap in prevention of intraoperative or postoperative retinal slippage. In present case, reattachment was obtained after initial MIVS. The postoperative complication of cataract formation has been developed in this case and no other complications such as subretinal perfluorocarbon, retinal slippage, recurrent retinal detachment, proliferative vitreoretinopathy formation or macular pucker needing additional surgery during follow up period of 19 months. Reviewing the literatures, Ando et al. introduced polyacetal retinal tacks together with conventional 20G vitrectomy for GRT with 81% of patients with retinal total or partial attached. However, MIVS has the advantage of smaller sclerotomy which might reduce the possibility of occurrence of intraoperative iatrogenic retinal breaks, enhance patient’s comfort and postoperative wound recovery. In addition, WAVs can improve the intraoperative field of view and offer better visibility and sharpness image of peripheral retina [5]. Currently, retinal tack had been abandoned by many surgeons due to unavailability and the concern of safety, we found it might be irreplaceable in cases of GRT with large inverted and rigid flap [8] and it may cause minimal or no retinal toxicity during as long as 21 years of follow-up [9]. To our knowledge, this is the first report of managing GRT with MIVS and retinal tacks in Taiwan.

## Conclusion

Although our report has limitation of small case number and relatively short follow-up period, our findings indicate that MIVS is a safe, effective and feasible method for managing GRT. In addition, in cases with large, inverted and rigid retinal flap, retinal tacks for stabilizing retinal flap might be inevitable to prevent retinal redetachment.

## Abbreviations

GRT: Giant retinal tear
MIVS: Microincisional vitrectomy surgery
PFCL: Perfluorocarbon liquids
WAVs: Wide-angle viewing systems
